# Antileukotrienes for the prevention and treatment of chronic lung disease in very preterm newborns: a systematic review

**DOI:** 10.1186/s12931-021-01800-1

**Published:** 2021-07-17

**Authors:** Marlide Jukema, Franciszek Borys, Greta Sibrecht, Karsten Juhl Jørgensen, Matteo Bruschettini

**Affiliations:** 1 Amsterdam UMC, Vrije Universiteit Amsterdam, Amsterdam, The Netherlands; 2 Poznan University of Medical Sciences, Poznan, Poland; 3 Department of Newborns Infectious Diseases, Poznan University of Medical Sciences, Poznan, Poland; 4 The Nordic Cochrane Centre, Rigshospitalet Dept., 7811, Blegdamsvej 9, 2100 Copenhagen, Denmark; 5 Cochrane Sweden, Dept. Research and Development, Skåne University Hospital, Clinical Science Lund, Lund University, Lund, Sweden

**Keywords:** Preterm infants, Chronic lung disease, Animal model, Respiratory morbidity, SYRCLE

## Abstract

**Background:**

Very preterm infants are at high risk of developing chronic lung disease, which requires respiratory support and might have long-term sequelae. As lung inflammation plays an important role in pathogenesis, antileukotrienes have been explored in both clinical and animal studies. We aimed to assess the benefits and harms of antileukotrienes for the prevention and treatment of respiratory morbidity and mortality in very preterm newborns.

**Methods:**

In this systematic review, we included randomized trials and non-randomized studies in humans and animals reporting the effects of antileukotrienes in very preterm infants or other mammals within 10 days of birth. Our pre-specified primary outcomes were all-cause mortality and any harm, and, for the clinical studies, incidence of chronic lung disease. Included studies underwent risk of bias-assessment and data extraction performed by two authors independently. There were no language restrictions.

**Results:**

Fifteen studies totally met our inclusion criteria: one randomized trial and four non-randomized studies in humans and 10 animal studies (five in rodents, two in lambs and one in either guinea pigs, rabbits or caprinae). All five clinical studies used montelukast and had a small sample size, ranging from 4 to 77 infants. The randomized trial (n = 60) found no difference in the incidence of chronic lung disease between the groups. Only one clinical study, which enrolled four very preterm infants and had a critical overall risk of bias, reported long-term outcomes. All other studies had unclear or greater overall risk of bias and meta-analyses were therefore deemed unfeasible. Eight of ten animal studies used leukotriene receptor antagonists as antileukotriene (montelukast in three of ten studies) and seven had an experimental study design (i.e. some animals were not exposed to antileukotrienes but no randomization). Three of the ten animal studies assessed different doses. Animal studies found no effect on the outcomes mortality, growth, or lung function related surrogate outcomes.

**Conclusions:**

Use of antileukotrienes in very preterm infants to prevent or treat chronic lung disease is not supported by the available evidence. Large randomized trials focusing on outcomes relevant to patients, including long-term outcomes, are needed. Studies should also minimize risk of bias.

## Background

Very preterm infants (born before 32 weeks’ gestational age) constitute an extremely vulnerable population and are at high risk of developing chronic lung disease [1]. Chronic lung disease is a broad term, which includes bronchial asthma and bronchopulmonary dysplasia (BPD). It has been reported that BPD is the most common complication in extremely preterm infants [2]. Defining BPD remains a challenge [3]. This is mainly due to there being multiple factors involved in the underlying pathophysiology. Injury to the lungs, both before and after birth, may lead to an abnormal reparative response. This could cause flawed lung development, which can affect lung function into adult life [2]. Caffeine is the only drug that reduces the rate of BPD [4], mortality, and neurodevelopmental disability [5]. More interventions are therefore needed to prevent and treat BPD and its consequences.

Antileukotrienes include leukotriene receptor antagonists (e.g. montelukast, zafirlukast and pranlukast) and leukotriene synthesis inhibitors (e.g. zileuton) [6]. Antileukotriene receptor antagonists (LTR As) bind competitively to cysteinyl leukotriene receptors 1 and block the contractile promoting activity of leukotrienes in airway smooth muscles.

Montelukast is the most common type in clinical use, is administered once a day, and can be taken without regard to meals [7]. Zafirlukast and pranlukast are administered twice a day. The LTRA s are processed mainly in the liver [8], metabolized mostly by CYP2C8, with the involvement of CYP2C9 CYP3A4 enzymes [9, 10]. It is worth mentioning that LTRAs are substrates for transporters [11] and the s of genes In children, common montelukast induced adverse events are headaches, abdominal pain, rash, thirst, hyperkinesia, asthma and eczema [13]. Pharmacovigilance studies have also reported increased frequency sleeping disorders in infants younger than 2 years and psychiatric disorders in children aged 2 to 11 years, being more frequently reported than in adults. This led to a US FDA alert being issued for psychiatric events being associated with montelukast. Eosinophilic granulomatosis may also be associated with the use of montelukast, but the role of LTRAs in its pathogenesis is still uncertain [15].

The drug zileuton, also an antileukotriene, has a different action mechanism from LTRAs. It works as an inhibitor of 5-lipoxygenase. The most serious concern is hepatotoxicity. Zileuton is mainly metabolized through the liver, particularly via P450 enzymes, mostly by CYP3A4 [16]. This can lead to problems when using drugs such as theophylline at the same time due to impaired metabolization of theophylline. An option is to halve the dose of theophylline when starting treatment with zileuton [8]. Theophylline is an example of a methylxanthine, which are known to have a protective effect on the respiratory system [17]. Methylxanthines are natural components of cocoa-based products and beverages such as coffee, tea and yerba mate and therefore are commonly present in the human milk, thus reaching the newborn.

The properties of antileukotrienes might have the potential to be useful in the prevention and treatment of chronic lung disease in very preterm infants and they are currently used clinically based on anecdotal evidence, though not approved for this purpose. Their harms and benefits have not been assessed systematically. This systematic review aims to explore the evidence base for antileukotrienes in very preterm neonates in both clinical and animal studies.

## Methods

Our methods for systematically reviewing the clinical studies are based on the template developed by the Cochrane Neonatal Review group (*Resources for Review Authors*, n.d.) [18]. Two separate protocols were registered in Prospero for the clinical and animal studies, respectively [19, 20], since Prospero requires authors to register separate protocols for clinical and animal studies. An exploratory pilot search for animal studies was performed before submitting the protocols and our comprehensive search and data extraction.

### Types of studies

We included randomized and non-randomized animal studies. Studies with a cross-over design were excluded due to our interest in long-term outcomes and the potential for carry-over effects.

For the clinical studies, we included randomized trials, quasi-randomized trials and non-randomized studies of intervention (NRSI). Again, we excluded trials with a cross-over design.

### Types of participants

We included studies in any neonatal mammals, both term and pre term. “Neonatal” was defined as the first 10 days since birth, which is an arbitrary cut-off point that we pre-specified in our protocol. For the clinical studies, we included very preterm infants with a gestational age below 32 weeks and who were admitted to a neonatal department.

### Types of interventions

For animal studies, we included studies using co-interventions and any route and dose of antileukotriene administration. We excluded studies where antileukotrienes were administered to mothers before birth or to lactating mothers. We also excluded studies where co-interventions were not available for all study arms. We included two types of studies (1) antileukotrienes versus control (either placebo, no intervention, or treatment as usual); (2) studies without any comparator (non-controlled studies).

For the clinical studies, we included two comparisons, i.e. (1) prevention and (2) treatment of chronic lung disease.

### Outcomes

#### Animal studies

Our primary outcomes for the animal studies were: (1) survival until last follow up; (2) any harm.

Our secondary outcomes were: (1) growth; (2) lung volume to body weight ratio; (3) lung function; (4) lung histology; (5) inflammation markers for lungs: levels of interleukins (IL), i.e. IL-1β, IL-6, IL-16, IL-8/CXCL-8, IL10, IL-4, IL-13, CC Chemokines (MCP-1, 1α, 1β, 2 and 3), Krebs von den Lungen (KL-6), Clara cell secretory protein (CC16), neutrophil gelatinase-associated lipocalin (NGAL), placental growth factor, N-terminal pro-BNP (NT-pro-BNP), macrophage migration inhibitory factor, NF-κβ, Soluble ICAM, Tumor Necrosis Factor-ά, cysteinyl leukotriene (cysLT) release in bronchoalveolar lavage fluid, airway eosinophilia, mucus hyperproduction; (6) lung injury; (7) a irway hyperresponsiveness, fibrosis and smooth muscle actin expression; (8) behavioral tests; (9) markers for apoptosis; (10) pulmonary vascular resistance, Fulton index, and arterial wall structure. We included animal studies regardless of outcomes. Most of these are surrogate outcome measures, which however might provide a useful insight on pathophysiology in exploratory animal studies.

#### Clinical studies

Our primary outcomes for the clinical studies were: (1) all-cause mortality during initial hospitalization; (2) BPD/chronic lung disease incidence: only for comparison one (i.e. prevention of chronic lung disease) according to the three definitions: [21–23]; (3) any harm.

Secondary outcomes were: (1) all-cause neonatal (first 28 days) mortality, only for comparison one (i.e. prevention of chronic lung disease); (2) retinopathy of prematurity (any and ≥ stage 3 [24]) (3) days of respiratory support; (4) days of supplemental oxygen; (5) need for mechanical ventilation (yes/no); (6) days of hospital stay; (7) major neurodevelopmental disability: cerebral palsy, developmental delay [25, 26] or Griffiths Mental Development Scale [27] assessment greater than two standard deviations (SDs) below the mean), intellectual impairment (intelligence quotient (IQ) greater than two SDs below the mean), blindness (vision less than 6/60 in both eyes), or sensorineural deafness requiring amplification. We pre-planned to assess data for children aged 18 to 24 months and aged three to five years separately; (8) each component of the composite outcome “major neurodevelopmental disability”; (9) pulmonary function test at school age (as specified by study authors).

### Searches

We searched the Cochrane Central Register of Controlled Trials (CENTRAL) in The Cochrane Library; MEDLINE via PubMed, and Embase, in September 2020. We also searched ongoing clinical trials submitted at clinicaltrials.gov and ITCRP website. We did not apply any restrictions regarding language, publication year, or publication status. Methodological filters excluding diagnostic studies were not used. Search strings for each database are listed in Appendix.

### Selection of studies

Two authors independently screened titles and abstracts and retrieved the full text of potentially relevant articles. Eligibility was assessed according to our inclusion criteria. Two authors independently performed data extraction and assessed risk of bias.

### Assessment of risk of bias

We used SYRCLE’s risk of bias tool [28] for animal studies, which include the following seven domains: selection bias due to sequence generation, baseline characteristics or inadequate allocation concealment; performance bias due to inadequate randomization housing or blinding; detection bias due to inadequate randomization of outcome assessment or blinding; attrition bias due to incomplete outcome data; reporting bias due to selective outcome reporting; and other sources of bias.

For non-randomized clinical studies, we used the ROBINS-I [29] tool to assess the risk of bias, which include the following eight domains: bias due to confounding; bias in selection of participants into the study; bias in classification of interventions; bias due to deviations from intended interventions; bias due to missing data; bias in measurement of outcomes; bias in the reported results; and the overall risk of bias. For the domain “confounding”, we took into account the following confounders: antenatal steroids, gestational age, birth weight, Apgar score, indication to start antileukotrienes and level of respiratory support at study entry.

For randomized trials, we used the Cochrane Risk of Bias 2 tool [30], which include the following five domains: bias arising from the randomization process; bias due to deviations of intended interventions; bias due to missing outcome data, bias in measurements of the outcome; bias in selection of the reported results and overall risk of bias.

Any disagreements were solved through discussion and, if necessary, by consulting a third review author.

### Data analysis

We planned to use the Cochrane software RevMan 5.4 [31] to synthesize and analyze data. We planned to analyze all infants and animals on an intention-to-treat basis and to use the fixed-effect model for meta-analyses because we expected a consistent treatment effect. We planned to synthesize data with risk ratios (RR) for dichotomous outcomes and mean differences (MD) for continuous outcomes, with 95% confidence intervals (CI). The overall certainty of the evidence was assessed using the Grading of Recommendations Assessment, Development and Evaluation (GRADE) approach, as outlined in the GRADE Handbook [32], for our primary outcomes.

### Subgroup analyses

For the animal studies, we planned the following: type of lung injury, dose and type of antileukotrienes.

For the clinical studies, we planned the following: (1) gestational age: extremely preterm infants (< 28 weeks’ gestation, very preterm infants (28 to 31 + 6 weeks’ gestation; (2) type of antileukotrienes: leukotriene receptor antagonists, leukotriene synthesis inhibitors; (3) age when first dose of leukotriene receptor antagonist was given; and (4) route of administration.

## Results

### Results of the search

**Fig. 1:**
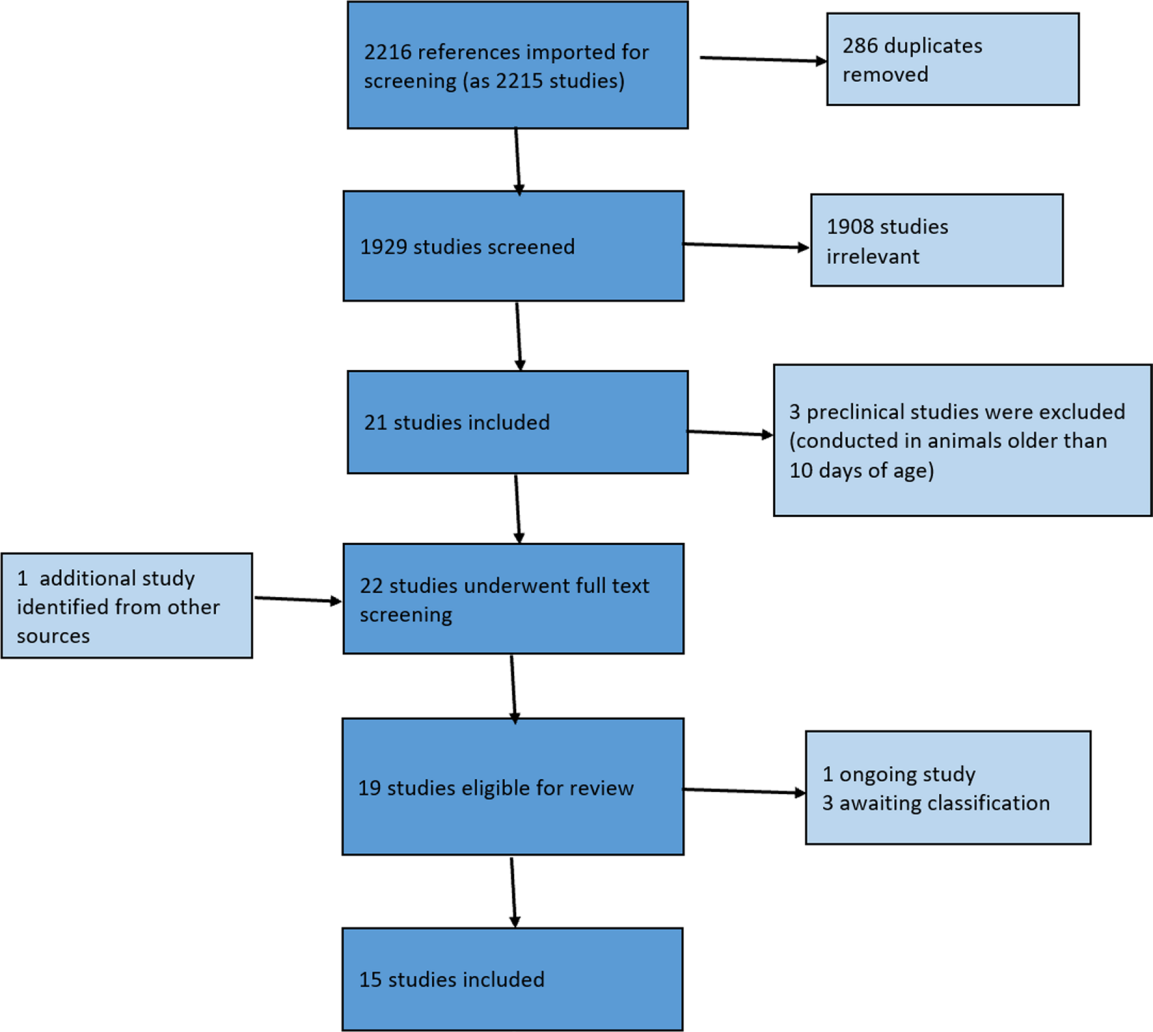
PRISMA flow diagram

Our searches for animal and clinical studies (Appendix) returned 1929 unique records. One additional study was identified through other sources (online search) while completing the review. Following screening titles and abstract, 22 studies were collected and assessed in full-text. Three animal studies were excluded because the animals were older than 10 days. Three studies were labelled as awaiting classification because the text of the conference abstracts were not available [33, 34] or because a protocol registered in 2007 was apparently not followed by a publication. One ongoing uncontrolled clinical study was identified, with a planned sample size of 200 very low birth weight newborns [35]. Thus, fifteen studies were included: ten animal studies (see Table 1) and five clinical studies of which one was a randomized trial (see Table 2). Figure 1 presents the PRISMA flow chart.

**Table 1:** Study characteristic animal studies

	Prevention studies (antileukotrienes are administered before inducing lung or systemic damage)		Studies on both prevention and treatment (antileukotrienes are administered before and after inducing lung or systemic damage)					Treatment studies (antileukotrienes are administered after inducing lung or systemic damage)		
	Demir 2008	Kertesz 1992	Cassin 1989	Phillips 1995	Schreiber 1985	Schreiber 1987	Xiao-Yan 2020	Chen 2018	Jouvencel 2003	Park 2011
Study design*	Experimental	Experimental	Observational	Experimental	Observational	Observational	Experimental	Experimental	Experimental	Experimental
Total number of animals at the very beginning	47	Not reported	24 lambs 10 goats	Not reported	6	16	72	45	24	Not reported
Number of animals which received antileukotrienes	Montelukast group n = 10 clarithromycin + montelukast + pentoxifylline combination group n = 6 (plus other study groups not relevant in this review)	Experiment 1: 31 0.1 uM/kg/h: 4 + 1 + 3 + 6 = 14 1.0 uM/kg/h: 8 + 9 = 17 experiment 2: not reported	Not reported	Prevention (normoxia): 19 (with three different doses) Treatment (hyperoxia): 22	5 (we use experiment FPL 57,231 infusion started during hypoxia, so exp 2)	5	24	Not reported	12	Not reported
Number of animals in control group	Clarithromycin n = 8 pentoxifylline n = 8 placebo n = 6	Experiment 1: 2 + 2 + 11 + 12 = 27 experiment 2: 60	No control group, i.e. all animals got antileukotrienes	Prevention (normoxia): 6 Treatment (hyperoxia): 6	No control group, i.e. all animals got antileukotrienes	No control group, i.e. all animals got antileukotrienes	24	Not reported	12	Not reported
Number of animals outcome data are reported for	47	Not reported	Sheep: 16 for antileukotrienes (plus 3 for thromboxane receptor antagonist) goats: 6 for antileukotrienes (plus 4 for thromboxane receptor antagonist)	Not reported	Not reported	Not reported	72	Not reported	16	Not reported
Funding	Supported by Dokuz Eylul University and The Scientific and Technological Research Council of Turkey	Antileuk provided by Stuart Pharmaceuticals Study partly supported by the Dee and Moody Research Fund of Evanston Hospital	National Hear, Lung and Blood institute Grant Antileukotrienes provided by the pharma	Supported by the Medical Research Council Drug provided by Upjohn Company	Supported in part by grants from the American Lung Association and U.S.Public Health Service Program Project Grant HL 24,056	Supported in part by grants from the American Lung Association, HL35518, and US Public Health Service Program Project Grant HL24056	Funded by Jiangsu Provincial Maternal and Child Health Research Project (F201647	Funding Project of Bengbu Medical College of Science and Technology Development (No. BYKF1741)	Grant sponsor: Société Française de Médecine Néonatale; Montelukast sodium a gift from Merck, Sharp and Dohme, Whitehouse Station, NJ	Medical Grant Program of Merck Sharp and Dohme Corp. (Rahway, NJ, USA), who also supplied with MK-0591 in powder form
Species	Rats	Rabbits	Sheep, goats	Guinea pig	Lambs	Lambs	Rats	Mice	Rats	Mice
Strain	Wistar	New Zealand albino	Not reported	Not reported	Mixed-breed	Not reported	Clean level P3 SD	57BL/6 J	Wistar	FVB/n
Age when antileuk/comparator were given	Postnatal days 3–13	day 7	Five days. Unclear, but it is likely that the animals were given antileukotrienes and were exposed to hypoxia at the same day	day 3–6	day 3–7	day 4–6	Not reported	day 2–14	day 4	Treatment windows were from days 1–4, 5–9 or 10–14 after birth
Presence and degree of prematurity	Full term	Full term	Not reported	Pre-term	Not reported	Not reported	Likely Full term	Likely Full term	Likely Full term	Full term
Mode of delivery	Naturally delivered	Not reported	Not reported	Caesarean section	Not reported	Not reported	Not reported	Not reported	Not reported	Naturally delivered
Type of lung damage/insult	Hyperoxia	Hyperoxia > 95% O_2_	Hypoxia, ventilation	Hyperoxia 95%O_2_	Hypoxia	LTD4 injection	Hypoxia	Hyperoxia	Hyperoxia 50% O_2_ from P0 to P15	Hyperoxia 85% O_2_
Age at lung damage	Days 3–13	Day 7	Five days. Unclear, but it is likely that the animals were given antileukotrienes and were exposed to hypoxia at the same day	days 1–3	Days 3–7	Days 4–6	Not reported	12 h, hyperoxia for 7 consecutive days	Within 24 h of birth	Within 24 h of birth
Type of control group	Clarithromycin Pentoxifylline Placebo	Vehicle	No control group, i.e. all animals got antileukotrienes	Vehicle	No control group, i.e. all animals got antileukotrienes	No control group, i.e. all animals got antileukotrienes	Saline for periventricular leukomalacia group	Saline (0.9% NaCl)	Saline	Vehicle (5% ethanol; 1% Tween 80)
Name of antileuk/name of comparator	Antileuk: Montelukast, Montelukast + pentoxifylline + clarithromycin combination Comparator: clarithromycin pentoxifylline	INTERVENTION ICI 198,615 (leukotriene receptor antagonist) CONTROL vehicle consisting of polyethylene glycol 400 (PEG 4OO), 1 M NaOH, and phosphate buffered saline (PBS)	Leukotriene receptor antagonist L 649923 Dual cyclooxygenease and lipoxygenase inhibitor BW 755C	U-75302 (LTB4 antagonist)	FPL57231 (leukotriene receptor antagonist)	FPL57231 (leukotriene receptor antagonist)	INTERVENTION Pranlukast CONTROL Saline	Antileuk: montelukast sodium Control: Saline	INTERVENTION Montelukast sodium CONTROL Normal saline	INTERVENTION MK-0591 (5-lipoxygenase-activating protein inhibitor) CONTROL vehicle
Dose	INTERVENTION montelukast: 1 mg/kg/day one dose combination: clarithromycin 100 mg/kg in two doses per day, montelukast 1 mg/kg/day, pentoxifylline 150 mg/kg in two doses per day CONTROL clarithromycin: 100 mg/kg/day in two doses pentoxifylline: 150 mg/kg/day in two doses saline: not reported	INTERVENTION experiment 1: two groups group 1: 0.1 uM/kg/h ICI group 2: 1.0 uM/kg/h ICI Experiment 2: 0.1 uM/kg/h CONTROL not reported	L 649,923: prepared in saline daily (10 mg/ml) and injected (5,86 mg/kg) over a 2 min period BW 755C: prepared in saline (8,8 mg/ml) and administered (30 mg/kg) over a 2- to 5-min period	3.0 mg/100 g body wt**	2 mg/kg/min (total 20 mg/kg)	2 mg/kg/min (total 20 mg/kg)	0.1 mg/kg	Montelukast 10 mg/kg Saline not reported	INTERVENTION 1 mg/kg/day (diluted in normal saline to 200mcg/ml—injected 5 mcg/g) CONTROL 5 mcg/g	INTERVENTION 40 mg/kg **
Frequency	INTERVENTION montelukast: 1 dose per day combination: clarithromycin in two doses per day, montelukast 1 dose per day, pentoxifylline in two doses per day CONTROL clarithromycin: in two doses per day pentoxifylline: in two doses per day saline: once daily	Continuous by micro-pump pumping 0.5 uL/h	We suspect that the drugs were only given once, but this cannot be extracted from the text with complete certainty	Every 12 h over a 72 h period	Once for 10 min	Once for 10 min	once every 12 h, for 3 consecutive days	Once every other day	1/day, from days 4–14	Once daily during the treatment window
Route of administration	INTERVENTION subcutaneously CONTROL clarithromycin: subcutaneously pentoxifylline: injected intraperitoneally saline: not reported	Subcutaneous pump	L 649,923: injected directly into the pulmonary circulation BW 755C: administered via femoral artery	Not reported	Infusion	Intravenous infusion	Intraperitoneal injection	Intraperitoneally	Subcutaneously	Subcutaneously

^*^Experimental: Compares outcomes with vs without antileuk administration (not all animals received antileuk); Observational: Compares outcomes before vs after antileuk administration (all animals received antileuk)

^**^The study reports results for different doses

None of the included studies reported on the following characteristics: protocol registration, immune status, sex, initiation dose

**Table 2:** Study characteristics clinical studies

	Cheng 2014	Kim 2015	Min Kim 2009	Panjwani 2016	Rupprecht 2014
Country	Taiwan	Korea	Korea	UK	Germany, USA
Protocol registration	Not reported	NCT01717625	Not reported	Not reported	DRKS00004763
Study design	Retrospective cohort	Multicenter, prospective, randomized, open labelled, parallel group, intervention trial	cohort study (preliminary investigation with the historical control group)	cohort study (all infants received antileukotrienes)	Unblinded, prospective trial (not-randomized)
Duration of follow-up	Unclear. At least two years based on info from Table 3 (MDI and PDI)	36 weeks GA, or the discharge date	12 weeks	Not reported	Treatment was continued until the radiological signs and the clinical symptoms of BPD disappeared or discharge
Completeness of follow-up	4/4	Intervention group: 30/37 Control group 36/40	15/15	13/13	intervention group: 10/11 (1 died) Control group: 4/11 (7 died)
Funding	Not reported	This study was supported by the research fund of the Korea Food and Drug Administration (KFDA)	Not reported	Not reported	Partly funded by an unrestricted grant from the Oberfrankenstiftung, Bayreuth, Germany, which had no influence on the design, collection, analysis, or interpretation of data or publication
Mode of delivery	Not reported	Not reported	INTERVENTION VD 4, C-Sect. 11 CONTROL VD 3, C-Sect. 12	Not reported	Not reported
Type of control group	No control group	Unclear	Standard treatment of BPD in the historical control group	No control group	Conventional therapy regimen
Total number of infants in intervention/control group	4	INTERVENTION 37 CONTROL 40	INTERVENTION 15 CONTROL 15	13	INTERVENTION 11 CONTROL 11
Gestational age	Ranging 24–30	INTERVENTION Mean 27.6 SD 1.6 CONTROL Mean 27.3 SD 1.6	INTERVENTION mean 27.3 SD 2.2 WEEKS CONTROL mean 27.1 SD 2.1 WEEKS	Mean gestation 25 + 3 weeks	INTERVENTION Mean 25.3 SD 1.6 CONTROL Mean 25.6 SD 1.3
Birth weight	Ranging 605–1490 g	INTERVENTION: 1,097 SD 327 CONTROL: 997 SD 235	INTERVENTION mean 913.7 SD 206.4 CONTROL mean 982.7 SD 260.1	Mean birth weight 746 g	INTERVENTION Mean 658 SD 138 CONTROL Mean 624 SD 144
Sex	Not reported	Not reported	INTERVENTION male 7, female 8 CONTROL male 7, female 8	Not reported	INTERVENTION male 7, female 4 CONTROL male, 7, female 4
Criteria (if any) to give intervention	Montelukast was given as rescue therapy when the patients’ chest X-rays showed fibrosis or increased infiltration; or when the patient required higher or prolonged ventilator support, which was defined as FiO2 ≥ 30%, PIP ≥ 20 cm H2O and ventilator usage more than nine days	< 32 weeks, > 14 days old on O2 or mechanical ventilation; > 20 cal/kg/day by enteral feeding	Existing BPD, admitted to the NICU except for cases where oxygen dependence other than lung diseases such as congenital anomalies, heart disease, and brain lesions may occur	“Last resort” in infants with significant oxygen requirement and radiological changes of significant lung disease unresponsive to postnatal steroids	Preterm infants with life-threatening BPD were chosen as the study group, with a probability of survival rated equal to or less than 50% by the attending physician. Further inclusion criteria for this study were a gestational age of less than 32 weeks, a birth weight of less than 1,500 g, and the need for mechanical ventilation support at day 28 after birth
Age when antileuk/comparator is given,	Not reported. Infants seem to be a few weeks old because of the reported body weight when the intervention was administered	INTERVENTION 31.3 SD 1.3 CONTROL 30.6 SD 1.6	Unclear	Not reported	Not clear. The recommended initiation of therapy was defined as the period between days 28 and 45 of life and as early as possible
Name of antileukotriene/comparator	Montelukast	Montelukast	Montelukast Sodium	Montelukast	Montelukast
Formulation	Singulair	Singulair	Singulair	Not reported	Not reported
Initiation	Not reported	Not reported	Not reported		1 mg/kg of body weight in the 1st week of therapy
Dose	2 mg	According to body weight (less than 1,000 g, 0.5 mg; 1,000 g to 1,500 g, 1.0 mg; 1,500 g to 2,000 g, 1.5 mg; greater than 2,000 g, 2 mg)	1 mg/kg	“2 mg/kg or 2 mg” (unclear reporting)	1 mg/kg of body weight in the 1st week of therapy, increasing to 1.5 mg/ kg of body weight in the 2nd week and finally to 2 mg/kg of body weight in the 3rd week
Frequency	Once daily for at least 28 days	Once daily until 36 weeks GA or until discharge	Twice a day for the average of 12 weeks	once daily	single dose, daily
Route of administration	Not reported	Orogastric tube or by oral administration	Orally	Orally	Not reported
Co-interventions	Not reported	Surfactant	Standard treatment for BPD	Not reported	All infants had varying concomitant medications administered (e.g. methylxanthines, steroids, and diuretics)

### Included studies

#### Animal studies

Of the ten included animal studies, five were in rodents (three in rats and two in mice) [36–40] and two were in lambs [41, 42]. The remaining three were in either guinea pigs [43], rabbits [44] or caprinae (sheep and goats in the same study) [45]. Two studies assessed prevention of respiratory morbidity only [36, 44], while three studied treatment effects only [37, 39, 40]. Five studied both prevention and treatment effects [38, 41–43, 45]. Eight of ten studies used leukotriene receptor antagonists such as antileukotrienes (montelukast used in three studies [36, 37, 39]), one studied a leukotriene synthesis inhibitor [40] and one studied both types of antileukotrienes [45]. Of the 10 animal studies, seven had an experimental study design (i.e. some animals were not exposed to antileukotrienes but were not randomized) and three an observational study design (i.e. all animals were exposed to antileukotrienes). Within three of the ten studies different doses were assessed [40, 43, 44].

#### Clinical studies

The five clinical studies included one randomized trial from Korea [46], a non-randomized study performed in Germany and the USA [47], and tree observational studies from Korea [48], the UK [49] and Taiwan [50]. Four studied treatment and one studied prevention of BPD [46]. All five assessed the same leukotriene antagonist; montelukast. The administered dose of montelukast ranged from 1 to 2 mg/kg body weight. Details are provided in Table 2.

### Risk of bias

#### Animal studies

Details of our risk of bias assessments are presented in Table 3. Overall, risk of bias was difficult to assess due to poor reporting and most domains were therefore “unclear” using the SYRCLE risk of bias tool [28]. As this tool is developed specifically for experimental animal studies, some domains were not applicable to the three non-controlled studies [41, 42, 45].

**Table 3:** SYRCLE risk of bias table

Study ID	Cassin 1989	Chen 2018	Demir 2008	Jouvencel 2003	Kertesz 1992	Park 2011	Phillips 1995	Schreiber 1985	Schreiber 1987	Xiao-Yan 2020
1. Selection bias- Sequence generation	Not applicable There is no control group, i.e. all the animals got antileukotrienes	Unclear risk Not reported	Unclear risk Not reported	Unclear risk Not reported	Unclear risk Not reported	Unclear risk Not reported	Unclear risk Not reported	Not applicable There is no control group, i.e. all the animals got antileukotrienes	Not applicable There is no control group, i.e. all the animals got antileukotrienes	Unclear risk Not reported
2. Selection bias- Baseline characteristics	Not applicable There is no control group, i.e. all the animals got antileukotrienes	Unclear risk Not reported	Unclear risk Not reported	Unclear risk Not reported	Unclear risk Not reported	Unclear risk Not reported	Unclear risk Not reported	Not applicable There is no control group, i.e. all the animals got antileukotrienes	Not applicable There is no control group, i.e. all the animals got antileukotrienes	Unclear risk Not reported
3. Selection bias- Allocation concealment	Not applicable There is no control group, i.e. all the animals got antileukotrienes	Unclear risk Not reported	Unclear risk Not reported	Unclear risk Not reported	Unclear risk Not reported	Unclear risk Not reported	Unclear risk Not reported	Not applicable There is no control group, i.e. all the animals got antileukotrienes	Not applicable There is no control group, i.e. all the animals got antileukotrienes	Unclear risk Not reported
4. Performance bias- Random housing	Not applicable There is no control group, i.e. all the animals got antileukotrienes	Unclear risk Not reported	Unclear risk The different groups were at least raised in the same room, but this does not report enough about the random housing	Unclear risk Not reported	Unclear risk Not reported	Unclear risk Not reported	Unclear risk Not reported	Not applicable There is no control group, i.e. all the animals got antileukotrienes	Not applicable There is no control group, i.e. all the animals got antileukotrienes	Unclear risk Not reported
5. Performance bias- Blinding	Not applicable There is no control group, i.e. all the animals got antileukotrienes	Unclear risk Not reported	Unclear risk Not reported	Unclear risk Not reported	Unclear risk Not reported	Unclear risk Not reported	Unclear risk Not reported	Not applicable There is no control group, i.e. all the animals got antileukotrienes	Not applicable There is no control group, i.e. all the animals got antileukotrienes	Unclear risk Not reported
6. Detection bias- Random outcome assessment	Unclear risk Not reported	Unclear risk Not reported	Unclear risk Not reported	Unclear risk Not reported	Unclear risk Random outcome assessment for one outcome; unclear for others	Unclear risk Not reported	Unclear risk Not reported	Unclear risk Not reported	Unclear risk Not reported	Unclear risk Not reported
7. Detection bias- Blinding	Not applicable There is no control group, i.e. all the animals got antileukotrienes	Unclear risk Not reported	Unclear risk Tissues were prepared in a blinded fashion, although there is no information about blinding all the outcome assessors	Low risk Quote: “All morphometric assessments were made blindly by the same observer (P.J.) (except for the bronchial alveolar attachments, by M.F.) on images of all lung sections”	Unclear risk Not reported	Unclear risk Not reported	Unclear risk Not reported	Not applicable There is no control group, i.e. all the animals got antileukotrienes	Not applicable There is no control group, i.e. all the animals got antileukotrienes	Unclear risk Not reported
8. Attrition bias- Incomplete outcome data	Unclear risk Unclear if outcome data are reported for all animals	Unclear risk Unclear if outcome data are reported for all animals	Low risk All animals were included in the analysis	Unclear risk Unclear if outcome data are reported for all animals	Unclear risk Unclear if outcome data are reported for all animals	Unclear risk Unclear if outcome data are reported for all animals	Unclear risk Unclear if outcome data are reported for all animals	Unclear risk Unclear if outcome data are reported for all animals	Unclear risk Unclear if outcome data are reported for all animals	High risk Quote "any loss of sample size due to deaths was made up for by random sampling"
9. Reporting bias- Selective outcome reporting	Unclear risk Protocol not available	Unclear risk Protocol not available	Unclear risk Protocol not available	Unclear risk Protocol not available	Unclear risk Protocol not available	Unclear risk Protocol not available	Unclear risk Protocol not available	Unclear risk Protocol not available	High risk Protocol not available; moreover, the study authors report that data for some outcomes are not shown	Unclear risk Protocol not available
10. Other- Other sources of bias	Low risk None relevant	Low risk None relevant	Low risk None relevant	Low risk None relevant	Low risk None relevant	Low risk None relevant	Low risk None relevant	Low risk None relevant	Low risk None relevant	Low risk None relevant

Legend of the colours in the table: green = low risk of bias, orange = unclear, red = high; grey = not applicable

The seven experimental animal studies all had unclear risk of selection bias because the randomization process and baseline characteristics were not specified. They all had an unclear risk of performance bias because none of the studies reported on random housing. The measures used to house the animals randomly within the animal room were not reported. Blinding of the investigators was also not reported. Only one study [37] was assessed as at low risk of detection bias as they reported that “all morphometric assessments were made blindly by the same observer (except for the bronchial alveolar attachments)”.

All studies had unclear or higher risk of reporting bias as their protocol was not available. One study reported that data for some outcomes were not shown [42] and thus had high risk of bias. The animal studies appeared free from other sources of biases.

#### Clinical studies

Details of our risk of bias assessments for the randomized and non-randomized studies are presented in Tables 4 and 5, respectively.

**Table 4:** Risk of Bias assessment with Rob 2.0 tool for the included RCT

	Randomization process	Deviations from intended interventions	Missing outcome data	Measurement of the outcome	Selection of the reported results	Overall risk of bias
Kim 2015	some concerns^a^	some concerns^b^	low^c^	some concerns^d^	low^e^	some concerns

^a^No information about allocation concealment, randomization of groups was performed using shuffled blocks of random numbers in Microsoft Office, Excel 2007

^b^Unclear description of the infants that were not included in the final analysis

^c^Data appears to be complete. Attrition and exclusions were explained (not completely clear though) and accounted for

^d^Unclear if outcome assessors were blinded

^e^Seems in accordance with protocol

**Table 5:** Risk of Bias assessment with ROBINS-I tool for the included non-randomized studies

	Confounding	Selection of participants into the study	Classification of interventions	Deviations from intended interventions	Missing data	Measurement of outcomes	Selection of the reported results	Overall risk of bias
Rupprecht 2014	Critical^a^	Low	Low	Low	Low	Moderate^f^	Low	Critical
Min Kim 2009	Low^b^	Serious^c^	Low	Moderate^d^	Moderate^e^	Moderate^g^	Moderate^h^	Serious
Panjwani 2016	No information	Serious^i^	No information	No information	No information	No information	No information ^j^	Serious
Cheng 2014	No information^k^	Critical ^l^	Moderate^m^	Low	No information	Moderate^n^	Moderate^o^	Critical

^a^The control group consisted of children whose parents provided informed consent for participation in this study (as a control group patient) but not for administration of the medication montelukast (controls 1–5, 8,and 9; Table 1); and children in whom the planned therapy scheme was not possible due to existing or arising contraindications for the study drug (4 children, phenobarbital therapy in controls 6, 7, 10, and 11)

^b^No significant difference between groups regarding patients' characteristics

^c^There is no clear definition of inclusion and exclusion criteria

^d^The study does not specify the exact time for which montelukast was given and for how long co-interventions of the conservative treatment were given, which may lead to relevant differences in co-interventions

^e^Data appears to be complete, although no protocol was published and the study was not registered as a clinical study

^f^Outcome "Duration for mechanical ventilation" might be biased by unblinded outcome assessor

^g^Outcome "Need for mechanical ventilation" might be biased by unblinded outcome assessor

^h^There is a discrepancy between text of the results section and table about vomiting or diarrhea as an adverse effect

^i^The study uses historical cohort as comparator, there is no clear definition of inclusion criteria, exclusion criteria are not well-specified

^j^Only abstract is available

^k^Information about possible confounding is insufficient

^l^Historical cohort, no clear definition of inclusion and exclusion criteria, no control group

^m^Subjective inclusion criteria

^n^Outcomes ‘hospital stay’ and ‘respiratory support (duration, days)’ are subjective

^o^No protocol published

None of the included clinical studies were assessed to have low risk of bias. The single included RCT had an overall risk of bias assessed as “some concerns” [46] due to missing information about the randomization process; unclear description of the infants that were not included in the final analysis and because it was unclear whether the outcome assessors were blinded. The cohort study did not clearly define inclusion and exclusion criteria and was therefore assessed to have serious risk of bias [48]. The study by Rupprecht et al. [47] was scored with an overall critical risk of bias because of confounding as the control group consisted of children whose parents provided informed consent for participation as a control group patient but not for administration of the medication montelukast. The reasons for only allowing the child into the control group are not reported. This leads to critical risk of bias in the domain ‘bias due to confounding’. The infants in the control group could have been potentially sicker than those in the montelukast group, in which case the parents might not be willing to try a drug with unknown effects on their fragile child. Therefore, the reduced rate of mortality in the infants treated with montelukast could be markedly different from the true effect. The study by Panjwani et al. [49] had a serious risk of bias. The study used a historical cohort as comparator and there was no clear definition of their inclusion and exclusion criteria. The study by Cheng et al. [50] had an overall critical risk of bias as a historical cohort design was used without clear inclusion and exclusion criteria.

### Effects of the interventions

Meta-analysis of the clinical and the animal studies was not deemed feasible for any of the outcomes since they were reported by too few studies with highly variable designs and were assessed with outcome measures which could not be meaningfully pooled.

#### Animal studies

Table 6 shows the list of the outcomes reported by each study.

**Table 6:** Outcomes antileukotrienes animals

	Chen 2018	Demir 2008	Jouvencel 2003	Kertesz 1992	Park 2011	Phillips 1995	Schreiber 1985	Xiao-Yan 2020
Mortality	Not reported	See note 1	See note 2	See note 3	See note 4	See note 5	Not reported	See note 6
Somatic growth	Not reported	See note 7	See note 8	Not reported	Not reported	Not reported	Not reported	Not reported
Lung volume to body weight	See note 9	Not reported	See note 10	See note 11	Not reported	Not reported	Not reported	Not reported
Lung histology	See note 12	See note 13	See note 14	Not reported	See note 15	See note 16	Not reported	Not reported
Inflammation markers for lungs	See note 17	Not reported	Not reported	See note 18	Not reported	See note 19	Not reported	Not reported
Lung injury	See note 20	Not reported	Not reported	Not reported	Not reported	Not reported	Not reported	Not reported
Airway hyperresponsiveness, fibrosis and smooth muscle actin expression	Not reported	See note 21	Not reported	Not reported	Not reported	Not reported	Not reported	Not reported
Behavioral tests	Not reported	Not reported	Not reported	Not reported	Not reported	Not reported	Not reported	See note 22
Pulmonary vascular resistance	Not reported	Not reported	Not reported	Not reported	Not reported	Not reported	See note 23	Not reported

None of the included studies reported on the following outcomes: Harms, lung function, markers for apoptosis, Fulton index, arterial wall structure

Schreiber 1987 and Cassin 1989 are not listed in the table as they reported none of the outcomes specified in our review

Intervention: montelukast 0/10 combination: 0/6 Control: clarithromycin 0/8 pentoxifylline 0/8 placebo 0/6

Intervention 0/12. Control 0/12

Experiment 1: not reported Experiment 2: intervention: the percent mortality of the rabbits at any given number of hours of exposure to > 95% (%) (48 h: 0; 60 h: 43; 84 h: 65 108 h: 88 132 h: 88). Control: experiment 2: The percent mortality of the rabbits at any given number of hours of exposure to > 95% (%) (48 h: 0; 60 h: 41; 84 h: 59 108 h: 79 132 h: 100). There were no significant differences at any time between the ICI and the control group

There was no mortality among study animals

In the prevention study (normoxia), 3 out of 19 and 1 out of 6 pups died in the antileukotriene and control group, respectively. In the treatment study (hyperoxia), 3 out of 22 and 0 out of 6 pups died in the antileukotriene and control group, respectively

This outcome cannot be calculated because “any loss of sample size due to deaths was made up for by random sampling

Intervention: montelukast: Me 13 SD 0.6 g; combination: Me 10.1 SD 1.1 g;:control clarithromycin: Me 9.3 SD 0.7 g; pentoxifylline: Me 9.2 SD 3.2; placebo: Me 11.6 SD 2.2 g; montelukast vs placebo p = 0.07; montelukast vs. clarithromycin p < 0.0001; montelukast vs. pentoxifylline p = 0.0019; combination vs. placebo p = 0.1661

Intervention: mean 28.8 SD 0.5(g) (not relevant); control: Mean 28.5 SD 0.4 g (not relevant)

LW/BW intervention: Not reported, → lung weight/body weight (LW/BW): It is impossible to extract the data due to wrong values on y-axis)

Intervention: Mean 5.3 SD 0.13(ml/100 g) (not relevant) control: Mean 5.15 SD 0.13 (not relevant)

LW/BW intervention: not reported(Lung water expressed as lung wet weight to body weight ratios 0.1 µM/kg/h ICI 48 h: 1.3 SD ?; 72 h: 5,7 SD 0.2; 84 h: 7.6 SD 0,4 96 h: 7.5 SD 0.5; 1.0 µM/kg/h ICI 84 h: 7.5 SD ?; 96 h: 6,6 SD 0.3). Control: lung wet weight: body weight ratios began to increase at 72 h and continued to increase slowly after 84 and 96 h of hyperoxic exposure. No differences between intervention and control group (Fig. 3b) Control Lung water expressed as lung wet weight to body weight ratios control 48 h: 1.7 SD ?; 72 h: 5.4 SD 0.2; 84 h: 6.2 SD 0.4 96 h: 6.3 SD 0.5

Intervention: mean linear intercept (MLI): 93 SD .5; radial alveolar count (RAC) mean: 4.28 SD 0.24—both p < 0.01 vs hyperoxia model. Control: mean linear intercept (MLI): 130 SD 7.7; radial alveolar count (RAC): 1.94 SD 0.1

Intervention: alveolar surface area (%): group 3 montelukast Me 41.6 SD 4.8; group 5 combination: Me 64.0 SD 3; control: alveolar surface area (%); clarithromycin Me 50.9 SD 4.2; pentoxifylline Me 59.4 SD 6.8; placebo Me 50.2 SD 10.4. montelukast vs. placebo p = 0.0389 montelukast vs. clarithromycin p = 0.0005 montelukast vs. pentoxifylline p < 0.0001 combination vs. placebo p = 0.0093

Intervention: surface density of parenchymal tissue mean 24.2 SD 1.2 (%) (not relevant); mean linear chord length mean 53.3 SD 1.3 (µm) (not relevant) septal attachments (/mm bronchi) mean 29.1 SD 1.0 (not relevant). Control: surface density of parenchymal tissue mean 22.8 SD 0.5 (not relevant); mean linear chord length mean 52.7 SD 1.3(not relevant) septal attachments (/mm bronchi) mean31.7 SD 0.9 (not relevant)

Number of airspaces intervention: treatment group: (dose 40 mg/kg, P10-14): mean 20 SD 2. Prevention group (dose 40 mg/kg, p1–4): mean 19 SD 1 control treatment group (dose 0 mg/kg, P10–14): mean 11 SD ? prevention: (dose 0 mg/kg, p1–4): mean 6 SD 2

95% oxygen + treatment: airspace (%) (37.0 SD 6.0) neutrophils (No mm^−2^) (198 SD 10.9 (Different from 95% O, control, p < 0.05)) lung sections from pre-term guinea pig pups. 21% oxygen + treatment: airspace (%) (43.5 SD 3.5) neutrophils (No mm^2^) (108 SD 8.5) lung sections from pre-term guinea pig pup

Intervention: relative TNF-α mRNA level mean: 2.0 SD 0.15; relative IL-6 mRNA level mean: 1.7 SD 0.06; relative IL-1β mRNA level: 1.9 SD 0.12; [not sure about p value, in the text: "Montelukast treatment significantly reduced the levels of TNF-a, IL-6, and IL-1b in the lung tissues of the BPD mice. control: relative TNF-α mRNA level mean: 3.3 SD 0.1; relative IL-6 mRNA level mean: 3.5 SD 0.2; relative IL-1β mRNA level mean: 2.9 SD 0.1

Intervention: Dose 0.1 μM/kg/h: Total protein recovered from BAL mean (µg/ml) (48 h and 72 h: 90 SD 20; 84 h: 250 SD 120; 96 h: 330 SD 40); PMNS represented as a percentage of the total (48 h: 0; 72 h: 1,3 SE 7; 84 h: 10 SE 5; 96 h: 18 SE 5) white cells recovered from BAL mean (%); PMNs, represented as the absolute number recovered from BAL of the left lung (× 100,000) (48 h and 72 h: 0.5 SE 0.2; 84 h: 2,4 SE 0.3 96 h: 2.9 SE 0.3); 6-Keto-PGF 1 alfa the stable metabolite of PGI, in pg/ml (48 h: 71 SE no info; 72 h: 54 SE 28; 84 h: 144 SE 50; 96 h: 347 SE 463); TXB, the stable metabolite of TXA, in pg/ml mean (48 h: 115 SE no info; 72 h: 81 SE 19; 84 h: 241 SE 121; 96 h: 207 SE 22). Dose 1.0 uM/kg/h: total protein recovered from BAL mean (µg/ml) (84 h: 475 SD 112; 96 h: 416 SD 56); PMNS represented as a percentage of the total (48 h: 0; 72 h: no info; 84 h: 20 SE 4; 96 h: 14 SE 5) white cells recovered from BAL mean (%); PMNs, represented as the absolute number recovered from BAL of the left lung (× 100,000) (48 h and 72 h: no info; 84 h: 2,9 SE 0.3 96 h: 2.1 SE 0.); 6-Keto-PGF, the stable metabolite of PGI, in pg/ml (48 h: no info; 72 h: no info; 84 h: 348 SE 32; 96 h: 315 SE 32); TXB, the stable metabolite of TXA, in pg/ml mean (48 h: no info; 72 h: no info; 84 h: 211 SE 19; 96 h: 259 SE 37)

Control: total protein recovered from BAL mean (µg/ml) (48 h and 72 h: 90 SD 20; 84 h: 392 SD 61; 96 h: 420 SD 56) PMNS represented as a percentage of the total (48 h: 0; 72 h: 1,3 SE 8; 84 h: 22 SE 5; 96 h: 21 SE 4) white cells recovered from BAL mean (%); PMNs, represented as the absolute number recovered from BAL of the left lung (× 100 000) (48 h and 72 h: 0.5 SE 0.2; 84 h: 3,4 SE 0,3 96 h: 3,5 SE 0,2); 6-Keto-PGF, the stable metabolite of PGI, in (48 h: 71 SE no info; 72 h: 54 SE 28; 84 h: 222 SE 32; 96 h: 265 SE 44) TXB, the stable metabolite of TXA, in pg/ml mean (48 h: 115 SE no info; 72 h: 81 SE 19; 84 h: 241 SE 121; 96 h: 207 SE 22) pg/ml

95% oxygen + treatment: neutrophil and eosinophil numbers and protein concentration in bronchoalveolar lavage fluid (BALF) neutrophils (10 4 ml-’ BALF) 3.0: 1.85 SD 0.79 (Different from equivalent vehicle control, PcO.05.)) eosinophils (10 6 ml -’ BALF) 3,0: 0.88 SD 0.37 protein (mg ml -’ BALF) 3,0: 0.28 SD 0.127). 21% oxygen + treatment: neutrophil and eosinophil numbers and protein concentration in bronchoalveolar lavage fluid (BALF) neutrophils(10 4 ml-’ BALF) 3,0: 1.45 SD 1.56 eosinophils (10 6 ml -’ BALF) 3.0: 0.94 SD 0.31(Different from equivalent vehicle control, PcO.05.) protein (mg ml -’ BALF)3.0: 0.27 SD 0.08)

Intervention: oxidative stress malondialdehyde 1.4 +—0.1 mcmol/g (mean, sd); SOD superoxide dismutase 22.0 +—1 IU/mg (mean, sd). Control: oxidative stress malondialdehyde 1.9 +—0.05 mcmol/g (mean, sd); SOD superoxide dismutase 16.5 + 1 IU/mg (mean, sd)

Degree of fibrosis absent /mild /moderate /marked Intervention: group 3 montelukast 0/1/6/3 group 5 combination: 4/2/0/0. Control: clarithromycin 0/1/3/4 pentoxifylline 2/2/4/0 placebo0/2/3/1. Actin score (density x intensity) Intervention: group 3 montelukast: 5 (2–9) group 5 combination: 0 (0–1) Control: clarithromycin 7.5 (2–9) pentoxifylline 1.5 (0–6) placebo 7 (2–12)

Compared with the PVL group, the escape latency of the rats in the Pran group was shortened (p < 0.05) (Table 2). On the 5th day of the experiment, there was a statistically significant difference in the number of times the rats in each group crossed the platform (F = 12.59, p < 0.001). Compared with the PVL group, the number of times (1.86 ± 0.23) of rats in the Pran group crossed the platform increased (p < 0.05)

Intervention: me 44.0 SD 7.0 in mmHg 1-1 min^−1^ kg^−1^. Control: me 70.3 SD 15.5 (p < 0.05 vs hypoxia + FPL 57,231) in mmHg1-1 min^−1^ kg^−1^ p = 0.0086

Four controlled studies reported on mortality and found no significant effect [36, 37, 40, 44]; two controlled studies reported on growth [36, 37]; no significant effect was found in either study between combination treatment (montelukast, clarithromycin and pentoxifylline combination) versus placebo.

We made the post hoc decision to include the reported outcome ‘lung weight to body weight ratio’, in addition to our prespecified outcome lung volume to body weight. No statistically significant difference was found in the three studies reporting on either of the two outcomes [37, 39, 44].

Five experimental studies assessed lung histology, reporting on different outcomes, i.e. radial alveolar count [39], alveolar surface area [36], parenchymal tissue [37], number of airspaces [40] and percentage of airspace [43]. No firm conclusions could be drawn (see Table 6 for more information).

Three studies reported on inflammation markers for lungs [39, 43, 44]. Two studies [43, 44] reported on polymorphonuclear leukocytes and protein in bronchoalveolar fluid. Phillips et al. [43] showed a reduction in the number of neutrophils and protein in the treated hypoxia group and in eosinophils in the treated normoxia group. The study by Chen et al. [39] detected a reduction in the concentration of other inflammation markers in the lung tissue of BPD mice.

Lung injury was reported in one study in which montelukast treatment decreased malondialdehyde levels and enhanced superoxide dismutase activity in the lung tissues of the BPD mice [39].

The study by Demir et al. [36] was the only study to report fibrosis and smooth muscle actin expression. They did not detect an effect of montelukast alone versus placebo; the combination treatment (montelukast, clarithromycin and pentoxifylline combination) did result in a lower actin score compared to the placebo group.

Only one study reported on behavioral tests, the Morris water maze experiment [38]. There was an improvement in escape latency in the pranlukast group and the number of times rats in the pranlukast group crossed the platform in the maze increased.

The study by Schreiber et al. [41] found a decrease in pulmonary vascular resistance in lambs after antileukotriene infusion.

None of the animal studies reported on harms, lung function, markers for apoptosis, Fulton index or arterial wall structure.

#### Clinical studies

Outcomes for the randomized trial [46] and the four non-randomized [47–50] clinical studies are reported in Table 7.

**Table 7:** Outcomes of the clinical studies

	Cheng 2014	Kim 2015	Min Kim 2009	Panjwani 2016	Rupprecht 2014
All-cause mortality (initial hospitalization)	0/6 Mortality seems not to be a prespecified outcome in this study, however no infants died	Not reported	INTERVENTION: 0/15 CONTROL: not reported	3/13 (2 had an antenatal history of oligohydramnios)	INTERVENTION 1/11 CONTROL 7/11
BPD definition (NIH/Jobe / Walsh/unclear)	Treatment study	Jobe INTERVENTION: mild 17/30; mod/severe 13/30 CONTROL: mild 17/36, mod/severe 19/36	NIH INTERVENTION: mild 4/15 moderate 5/15 severe 6/15 CONTROL: mild 5/15 moderate 3/15 severe 7/15	Treatment study	Treatment study
Harms	Not reported	INTERVENTION / CONTROL Infection: 8/3 Gastrointestinal disorders: 5/1 Blood and lymphatic system disorders: 2/1 Cardiac disorders: 1/0 General disorders and administration site conditions: 2/1 Hepatobiliary disorders: 1/0 Pregnancy, puerperium and perinatal conditions: 1/0 Renal and urinary disorders: 2/0 Respiratory, thoracic and mediastinal disorders: 1/1 Vascular disorders: 1/0 Investigations: 4/6	INTERVENTION/ CONTROL Fever: 0/0 Diarrhea: 1/2 Cough: 0/0 Dermatatis: 0/0 Hypersensitivity reactions: 0/0 Vomiting symptoms: 0/0 t	“ No obvious side effects were noted”	No drugs- associated adverse events were identified; unclear about other adverse events
Hospital stay	ranging 98–138 days				
All-cause neonatal mortality	0	Not reported	Not reported	Not reported	Treatment study, no info about the time of drug administration
Respiratory support (duration, days)	ranging 7–77 days	Not reported	Not reported	mean ventilation days 41.4 (range 7–69)	INTERVENTION: mechanical ventilation time: mean 41.2 SD 25.3 days CONTROL: mechanical ventilation: 103.7 SD 90.6 days
Need for mechanical ventilation	Not reported	INTERVENTION: 7/37 at 2 weeks CONTROL: 7/40 at two weeks	INTERVENTION: before intervention: 11/15 after 2 weeks of montelukast 7 CONTROL: before intervention: 11; after 2 weeks of montelukast 8	Not reported	Not reported
Major neurodevelop-mental disability	Mental developmental index at two years old: ranging 76–108 PDI at two years old: ranging 96–114				
Retinopathy of prematurity	1	Not reported	Not reported	8	Not reported

None of the included studies reported on the following outcomes: FiO_2_ (duration, days), pulmonary function testing at school age

Two clinical studies reported on all-cause mortality [47, 48]; only one study [47], non-randomized, reported on all-cause mortality for both the intervention and the control group and found a significant reduction in all-cause mortality in the montelukast group.

The two studies that reported on frequency and the severity classification of BPD showed no relevant difference between case and control group [46, 48].

The occurrence of adverse events did not differ between intervention and control groups in either the randomized trial [46] or the observational study by Kim [48]. It was unclear whether the other three studies had planned to report adverse events, but they did not.

Rupprecht et al. [47] did not provide information about the timing of drug administration and therefore all-cause neonatal mortality could not be extracted from the study for our pre-defined time point. Kim et al. [46], Panjwani et al. [49] and Min Kim et al. [48] did not report all-cause neonatal mortality.

Rupprecht et al. [47] reported a significantly shorter duration of respiratory support in the group receiving montelukast compared to controls (41.2 ± 25.3 vs. 103.7 ± 90.6 days).

Two studies reported on mechanical ventilation and found no differences [46, 48].

None of the included studies reported on fraction of inspired oxygen duration or pulmonary function testing at school age.

### GRADE assessment

The certainty of the evidence was “very low” for all outcomes because of imprecision and high risk of bias in multiple other domains, both in clinical and animal studies.

## Discussion

### Summary of main findings

In this systematic review, we aimed to systematically assess the effects of antileukotrienes for the prevention and treatment of chronic lung disease in very preterm newborns. We included five clinical studies and ten animal studies. The clinical studies consisted of one RCT and four non-randomized studies. These five clinical studies and three of the animal studies examined the leukotriene antagonist montelukast. We did not find it meaningful to pool results because of the differences in study design and the high overall risk of bias. Drawing definitive conclusions on basis of the existing evidence is thus not possible.

### Overall completeness and applicability of evidence

The animal studies had an overall unclear risk of bias due to poor reporting. None of the experimental studies reported on sequence generation, baseline characteristics, allocation concealment, random housing, blinding of the caregivers or random outcome assessment. Only Jouvencel [37] reported adequately on blinding of the outcome assessor, and Demir [36] on completeness of the outcome data, whereas the other studies lacked information. The fact that a protocol was not available for any of the animal studies is also noteworthy. This leads to unclear risk of reporting bias and poor transparency in general. In the case of Schreiber [42] it was also mentioned that data for some outcome was not shown, which causes a high risk of bias for outcome reporting. We classified Phillips [43] as assessing both prevention and treatment effects, as we considered the pups treated with antileukotriene in normoxic conditions as the prevention group and the pups with hyperoxia as the treatment group. Seven studies had an experimental design, i.e. the animals were exposed to two or more different interventions, whereas in the remaining three studies all animals received the same intervention and were therefore defined as observational.

Only one study reported outcome data following hospital discharge [50]. Kim et al. [46] is the first prospective study of montelukast for very preterm infants. Min Kim [48] was a cohort study with a historical control group. The study did not provide a clear definition of the inclusion and exclusion criteria, which leads to a serious risk of bias in the selection of participants.

### Relation to other research

A study from 2019 evaluated incidence trends of neonates born very preterm in 11 high-income countries and reported increased BPD rates in most countries [51]. Main reasons for this trend include the increased survival of extremely preterm infants and active resuscitation at lower gestational age. Additional interventions are needed to prevent and treat this condition. Of note, montelukast is already being used as a drug in infants with BPD [52]. Interestingly, in this leaflet released/published by American Thoracic Society, montelukast is only listed as an anti-inflammatory medicine for children with BPD.

The administration of off-label drugs in neonates are a universal problem. This forces the neonatologist to rely mostly on clinical experience, expert consensus and data extrapolation from patients other than neonates when deciding upon drug choice and dosage [53]. This supports the need for additional high quality research on this topic. We identified one ongoing observational study that aims to explore the effects of montelukast on very low birth weight infants with BPD [35]. The planned sample size, 200 infants, is considerably larger than the clinical studies performed so far and might allow to better explore potential harms of antileukotrienes administration. However, a randomized design would be preferable to assess the efficacy.

### Strengths and weaknesses of our review

This is the first systematic review that explores the evidence base of antileukotrienes in very preterm infants in both clinical and animal studies. The review has several strengths. We conducted a comprehensive search with no date or language restrictions. We had studies translated from Mandarin [38] and Korean to English [48]. Further, all the potentially eligible titles and abstracts were screened independently by two authors, as were data extraction and the assessment of risk of bias. We used the most recent and validated tools to assess risk of bias in trials, non-randomized studies and animal studies.

Limitations include our arbitrary definition of neonate animals, i.e. up to 10 days of life. As the definition of a newborn infant (up to 28 days of life) is not based on a specific developmental phase or level of maturation it is not possible to identify a corresponding age in animal models. Further, we did not find any meta-analyses feasible. To retrieve additional information we contacted the authors of two conference abstracts and of the registered protocol we identified in our searches, however, we did not receive any response. Therefore, we could not include these studies and this restriction, though outside of our control, is a potential source of bias.

### Implications for research and practice

Refining the existing models to recapitulate the pathology at play in the infants is an urgent matter in order to better evaluate new interventions for BPD [54]. Most animal experiments are carried out to gather information about health in humans and aim to investigate new interventions that are intended for future use in humans. Differences in outcomes in animals and humans are partly due to fundamental biological differences. However, other factors such as for instance design, conduct and reporting play an equally important role [55]. Future animal studies should be designed with higher quality and aim to minimize potential sources of bias, as described in the SYRCLE tool [28]. The registration of the protocols of animal studies in free databases such as https://preclinicaltrials.eu/ should become a standard practice and become a formal requirement placed by journals to publish such studies, as is already commonly done for the clinical studies. Similarly, an appropriate randomization should be performed to ensure that animals in each group are in the same housing conditions (e.g. temperature, humidity, light, noise, odors) and to avoid that researchers subjectively select which animals and samples to be used for outcome assessment. Finally, animal studies should clearly report how many animals were used in each step of the experiment, from inclusion to reporting of all outcomes, so that attrition bias can be assessed. As all the animal studies included in this review failed to address these key components in conducting and reporting, the translational value is extremely limited.

Seven of the ten animal studies used an antileukotriene other than montelukast. We speculate that, unless justified by species or pharmacokinetics characteristics, in future animal studies only montelukast should be administered, as only this drug has been used in all clinical studies so far, including the large ongoing study. When choosing outcomes, the focus should be on those with clinical relevance, such as mortality, improved respiratory function and harms.

Future clinical studies should preferably be designed as large, high quality RCTs [56]. New trials are necessary as the harms are not negligible. The findings of the ongoing study [35] with a planned sample size of 200 infants are not available yet. Multicenter RCTs would be an option in order to reach a sufficient sample size. Just as for animal studies, the focus should be on clinically relevant outcomes.

## Conclusions

Based on the available evidence, no reliable conclusions about the clinical relevance of antileukotriene administration to very preterm infants can be drawn. Large randomized trials that focus on outcomes relevant to patients and their families, including long-term outcomes, are needed. Animal studies should prioritize montelukast over other antileukotrienes and minimize risks of bias.

## Data Availability

The data are available by accessing the published studies listed in Tables 1 and 2.

## Abbreviations

BPD: Bronchopulmonary dysplasia
CI: Confidence interval
IL: Interleukin
IQ: Intelligence quotient
LTRA: Antileukotriene receptor antagonist
MD: Mean difference
NICU: Neonatal intensive care unit
NRSI: Non-randomized study of intervention
RR: Risk ratio
SD: Standard deviation
GRADE: Grading of Recommendations Assessment, Development and Evaluation
